# Deciphering the role of CX3CL1-CX3CR1 in aortic aneurysm pathogenesis: insights from Mendelian randomization and transcriptomic analyses

**DOI:** 10.3389/fimmu.2024.1383607

**Published:** 2024-04-23

**Authors:** Xingyu Qian, Yidan Zheng, Li Xu, Zongtao Liu, Ming Chen, Fuqiang Tong, Pengning Fan, Zhe Chen, Nianguo Dong, Chao Zhang, Junwei Liu

**Affiliations:** Department of Cardiovascular Surgery, Union Hospital, Tongji Medical College, Huazhong University of Science and Technology, Wuhan, Hubei, China

**Keywords:** aortic aneurysm, Mendelian randomization, single-cell RNA sequencing, cytokine, inflammation

## Abstract

**Background:**

The crucial role of inflammation in aortic aneurysm (AA) is gaining prominence, while there is still a lack of key cytokines or targets for effective clinical translation.

**Methods:**

Mendelian randomization (MR) analysis was performed to identify the causal relationship between 91 circulating inflammatory proteins and AA and between 731 immune traits and AA. Bulk RNA sequencing data was utilized to demonstrate the expression profile of the paired ligand-receptor. Gene enrichment analysis, Immune infiltration, and correlation analysis were employed to deduce the potential role of CX3CR1. We used single-cell RNA sequencing data to pinpoint the localization of CX3CL1 and CX3CR1, which was further validated by multiplex immunofluorescence staining. Cellchat analysis was utilized to infer the CX3C signaling pathway. Trajectory analysis and the Cytosig database were exploited to determine the downstream effect of CX3CL1-CX3CR1.

**Results:**

We identified 4 candidates (FGF5, CX3CL1, IL20RA, and SCF) in multiple two-sample MR analyses. Subsequent analysis of the expression profile of the paired receptor revealed the significant upregulation of CX3CR1 in AA and its positive correlation with pro-inflammatory macrophages. Two sample MR between immune cell traits and AA demonstrated the potential causality between intermediate monocytes and AA. We finally deciphered in single-cell sequencing data that CX3CL1 sent by endothelial cells (ECs) acted on CX3CR1 of intermediated monocytes, leading to its recruitment and pro-inflammatory responses.

**Conclusion:**

Our study presented a genetic insight into the pathogenetic role of CX3CL1-CX3CR1 in AA, and further deciphered the CX3C signaling pathway between ECs and intermediate monocytes.

## Introduction

Aortic aneurysm (AA), characterized by pathological dilations of the aorta, constitutes a significant cardiovascular morbidity and mortality risk (1). Predominantly localized within the abdominal or thoracic segments, aortic aneurysm presents a complex interplay of various pathogenic mechanisms. Multiple stimuli lead to phenotypic changes in smooth muscle cells and ultimately to degradation of elastic fibers and collagen deposition (2). Of particular relevance is the burgeoning understanding of the pivotal role of inflammatory cascade.

The inflammatory response in AA is orchestrated by a spectrum of cytokines and immune cell infiltration. Recent studies demonstrated the accumulation of CD4^+^ T cells, monocytes, macrophages, and B cells in the dilated arterial wall (3, 4). In parallel, several cytokines including CCL5, HBD1, and ICAM1 were confirmed in association with AA (5). However, such endpoint-driven conclusions could only identify the potential of these targets as biomarkers, as it is challenging to characterize whether the appearance of these immune cells/inflammatory cytokines is a cause or a consequence of AA, which is one of the reasons why there is a lack of effective pharmacological interventions.

Mendelian Randomization (MR) emerges as a potent genetic epidemiological approach (6), poised to discern the causal relationship between inflammation and aortic aneurysms. With the advances in genomics and large cohort studies, over 20 potential risk loci for AA were established (7), but two-sample or multi-sample MR studies to decode the pathological risk for AA are still lacking.

Here we aimed at elucidating the causal relationship between circulating cytokines/immune cells and AA. With the assistance of bulk and single-cell RNA sequencing data, we identified the key role of the CX3CL1-CX3CR1 axis in CD14^+^CD16^+^ monocytes in the pathogenesis of AA.

## Materials and methods

### Study design

**Figure 1:**
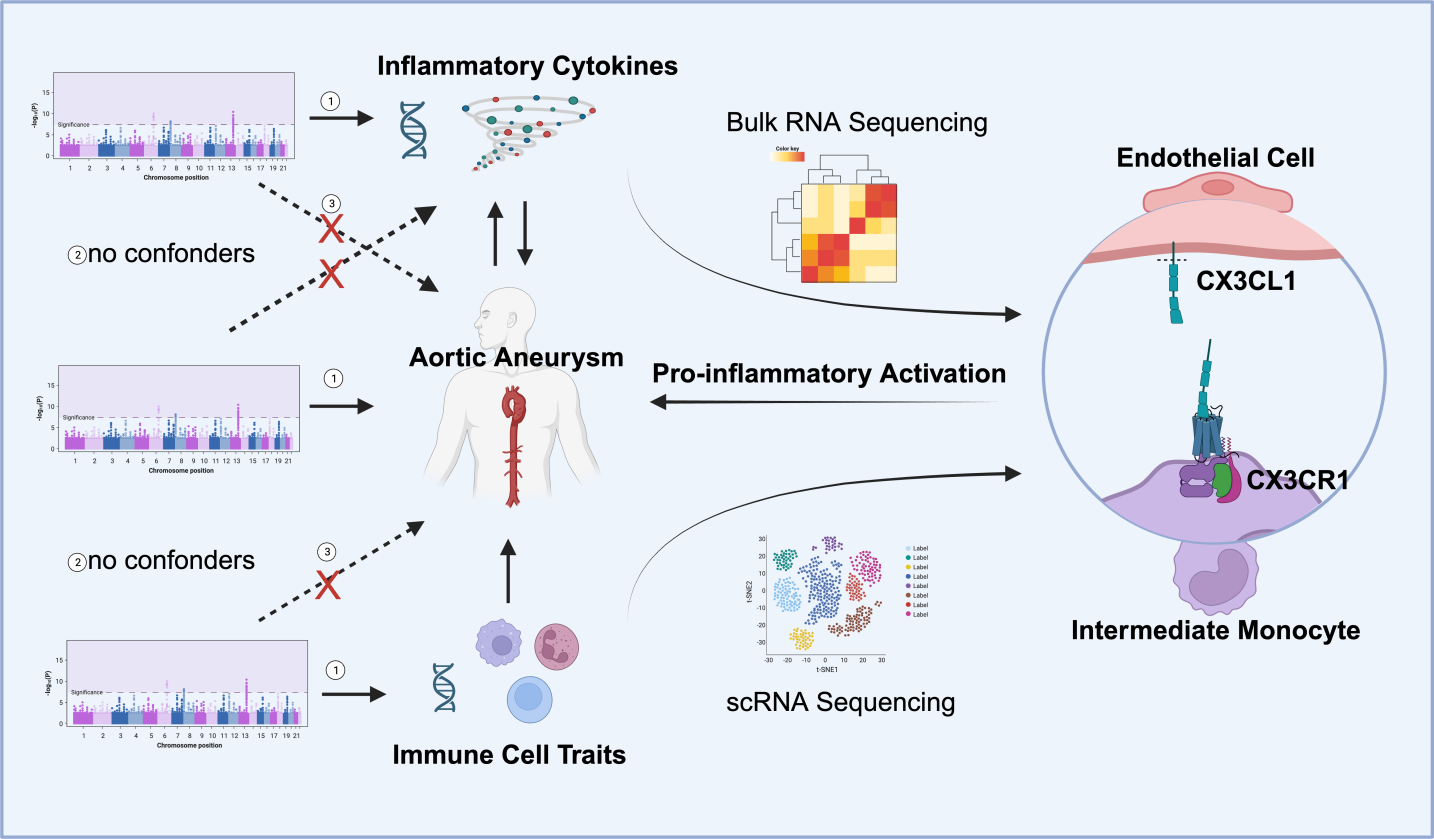
Overall study design. Mendelian randomization (MR) analyses identified the reciprocal causality between circulating inflammatory cytokines and AA and the causality between immune cell traits and AA. Screened candidates CX3CR1 and CX3CL1 were analyzed by transcriptomics, and the ligand receptor interaction between endothelial cell and intermediate monocyte was found to lead to pro-inflammatory activation and promote the development of aortic aneurysm (Images created with BioRender.com). Three hypotheses of the MR study include 1. Genetic variation as an instrumental variable is associated with exposure; 2. Genetic variation is not associated with confounding factors; 3. Genetic variation affects outcomes only through exposure and not through other pathways.

We used published genome-wide association studies (GWAS) summary data for 91 circulating inflammatory cytokines, 731 immune cells, and aortic aneurysm (AA). In our study, multiple two-sample MR analysis was carried out in order to investigate causality between inflammatory cytokines and AA and between immune traits and AA. Meanwhile, the reverse MR method was conducted to explore the potential reverse causal effects between AA and inflammatory cytokines. The MR analysis was based on three key assumptions: 1. the genetic variants selected as IVs were truly associated with the exposure; 2. the genetic variants had to be independent of any confounding factors; 3. the genetic variants affected the outcome only through exposure. Bulk RNA sequencing data and single-cell RNA sequencing data were utilized to validate the MR findings. The study design overview is presented in **Figure 1**.

### GWAS data source

GWAS summary statics for AA were obtained from the GWAS Catalog (accession number GCST90018783). A total of 3,230 European ancestry cases, 475,964 European ancestry controls (Finngen and UK Biobank), 1,155 East Asian ancestry cases, and 173,601 East Asian ancestry controls (Biobank Japan) were included (8).

GWAS summary statistics for the level of 91 circulating cytokines were derived from the largest and latest available genome-wide protein quantitative trait locus (pQTL) study, which covers 14824 participants from 11 independent cohort studies (9).

GWAS summary statistics for each immune trait are publicly available from the GWAS Catalog (accession numbers from GCST0001391 to GCST0002121). A total of 731 immunophenotypes including absolute cell (AC) counts (n = 118), median fluorescence intensities (MFI) reflecting surface antigen levels (n = 389), morphological parameters [MP] (n = 32) and relative cell (RC) counts (n = 192) were included. The original GWAS on immune traits was performed using data from 3,757 European individuals and there were no overlapping cohorts (10).

### Instrumental variables selection

Firstly, we set a threshold of *p*<5×10^-8^ to screen SNPs strongly related to inflammatory cytokines, immune traits, and AA, however, quite a few SNPs were screened out. Then we applied a genome-wide significance threshold of *p*<5×10^-6^. Secondly, we clumped SNPs with the criteria where kb = 10000, and *r^2^ =* 0.001 to avoid linkage disequilibrium (LD). Thirdly, the bottom line of F-values for each SNP was set to 10 to avoid weak instrumental bias (11).

### MR statistical analysis

MR analysis is conducted utilizing the “TwoSampleMR” R package (Version 0.5.6) of the RStudio (version 4.2.3). Inverse variance weighting (IVW) was employed as the main method, with MR Egger regression, weighted median, and simple median as complementary methods. MR results were ultimately identified as statistically significant only if they met with the following conditions: 1. the 95% confidence intervals (CI) derived from IVW did not cross over 1, 2. *p*<0.05, 3. IVW results met the consistent trend of effects derived from the other three MR analyses.

We employed three sensitivity analysis methods to evaluate the sensitivity of MR results, including the heterogeneity test, pleiotropy test, and leave-one-out sensitivity test. Cochran’s Q test was used to determine the heterogeneity, and *p*>0.05 was taken as no heterogeneity. The intercept of the MR Eggar analysis results was used to evaluate the horizontal pleiotropy, with *p*>0.05 considered as no pleiotropy. Leave-one-out sensitivity was used to investigate whether the causal association was influenced by SNP shared between exposure and outcome.

### Transcriptional data acquisition and processing

Transcriptional profiles of AA were obtained from the GEO dataset with the accession number GSE141032 containing 12 AA samples and 12 organ donor samples utilized as controls. The raw counts from the original matrix were processed with R package “DESeq2” to obtain the normalized counts and significantly differentially expressed genes (DEGs) with p<0.05 and log2 fold change (FC) >0.95.

### Single cell sequencing data acquisition and processing

Previously published single cell sequencing (scRNA-seq) data from control (n = 3) and ascending thoracic aortic aneurysm cases (n = 8) were included for reanalysis (3). Cells were removed under the condition of expressing fewer than 200 genes or greater than 20% mitochondrial genes. R package Seurat (version 4.2.0) were used to perform dimensional reduction of scRNA-seq data. The “NormalizeData” and “ScaleData” function from Seurat was used for normalization and the scale factor was set to 100,000, then followed by “FindVariableFeatures” with default parameters to calculate highly variable genes for each sample. “FindIntegrationAnchors” in the Seurat package were applied to remove batch effect and merge samples. Top 2 000 genes with the highest expression and dispersion from each sample were determined as the integration anchors and used for data integration. Then, cells were projected in 2D space using Uniform Manifold Approximation and Projection (UMAP). To identify differentially expressed genes (DEGs) among each cluster, the “FindAllMarkers” function from Seurat was used and non-parametric Wilcoxon rank sum tests were set to evaluate the significance of each individual DEG. DEGs with adjusted P value less than 0.05 were thought to be significant and used in downstream analysis. Hierarchical clustering and heatmap generation were performed for single cells on the basis of normalized expression values of marker genes curated from the literature or identified significant DEGs. “DimPlot” and “VlnPlot” were used to visualize the expression of individual genes, cells were grouped by their cell type as determined by analysis with Seurat.

The trajectory analysis based on gene expression changes among the cell clusters was performed using R package Slingshot (version 2.1.0.0). We infers the key elements of the global lineage structure using a minimum spanning tree method based on clustered cells. The start point of trajectories was defined based on DEGs.

CellChat analysis was performed by R package CellChat (version 1.6.1). Function ‘createCellChat’ was selected to create a CellChat object for the overall gene expression matrix, and then the cell annotation information identified by scMGCA was added through the function ‘addMeta’. CellChatDB.human provided by CellChat was used for the human ligand receptor reference. Finally, the cell-cell communication probability is inferred using the function ‘computeCommunProb’, and the communication probability at the level of each cell signaling pathway is inferred by the function ‘computeCommunProbPathway’. The figure is generated with the function ‘netVisual_aggregate’.

### Functional enrichment analysis

Gene Ontology (GO), and Kyoto Encyclopedia of Genes and Genomes (KEGG) pathway analyses were performed to. The R package “org.Hs.eg.db” was used to obtain the Entrez ID for each DEG, and “clusterProfiler” was employed for biological function analyses.

### Estimation of immune cell infiltration

Cell-type identification by estimating relative subsets of RNA transcripts (CIBERSORT) was utilized to deconvolute gene expression into immune cell fractions (12). Spearman correlation analysis was conducted to determine the correlation between the expression of CX3CR1 and infiltrated immune cells.

### Human specimen collection and immunofluorescence staining

Aortic wall tissue was taken from patients undergoing aortic replacement surgery due to AA and the recipient undergoing heart transplantation due to ischemic cardiomyopathy at Wuhan Union Hospital, Wuhan, China. The specimens were fixed in 4% paraformaldehyde immediately after separation from the body. The Review Board of Union Hospital, Affiliated to Tongji Medical College, Huazhong University of Science and Technology, Wuhan, China, approved the collection of human aorta tissues and their use in our study. The Declaration of Helsinki’s guiding principles were followed when conducting the research. Written informed permission was obtained from each recruited patient for the use of their aorta tissue in the study. Immunofluorescence staining was performed as previously described (13).

## Results

### The causal effect of genetically predicted circulating cytokines on AA

**Figure 2:**
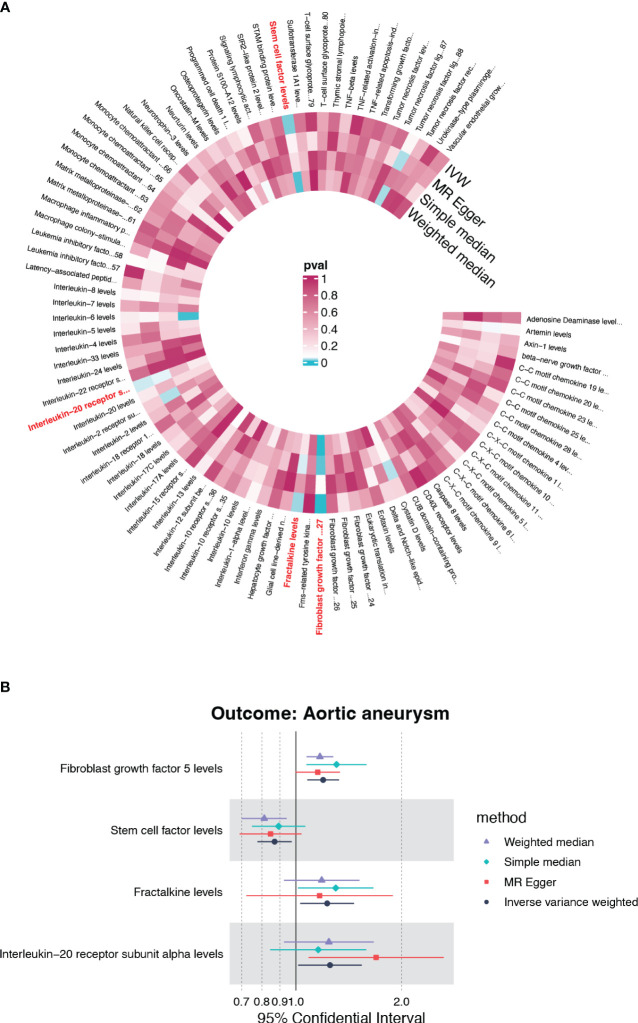
The causality of inflammatory cytokines on AA. **(A)** Cyclic heatmap demonstrating p-values for 91 cytokines with each of the four methods (IVW, MR Egger, Simple median and Weighted median). Preliminary candidates (*p*<0.05 with IVW) are marked in red. **(B)** Forest plot illustrating ORs and 95% CIs for FGF5, SCF, Fractalkine and IL20RA as exposures separately.

Under the threshold of *p*<5×10^-6^, fibroblast growth factor 5 (FGF5), stem cell factor (SCF/c-kit), Fractalkine/CX3CL1 and Interleukin-20 receptor subunit alpha (IL20RA) were first screened out of the 91 genetically determined circulating inflammatory cytokines based on the IVW method (**Figure 2A**). Among the four candidates, FGF5, CX3CL1, and IL20RA were associated with an increased risk of AA, and c-kit was associated with a reduced risk of AA (**Figure 2B**).

### Identify the causal effect of FGF5, CX3CL1, IL20RA and SCF

**Figure 3:**
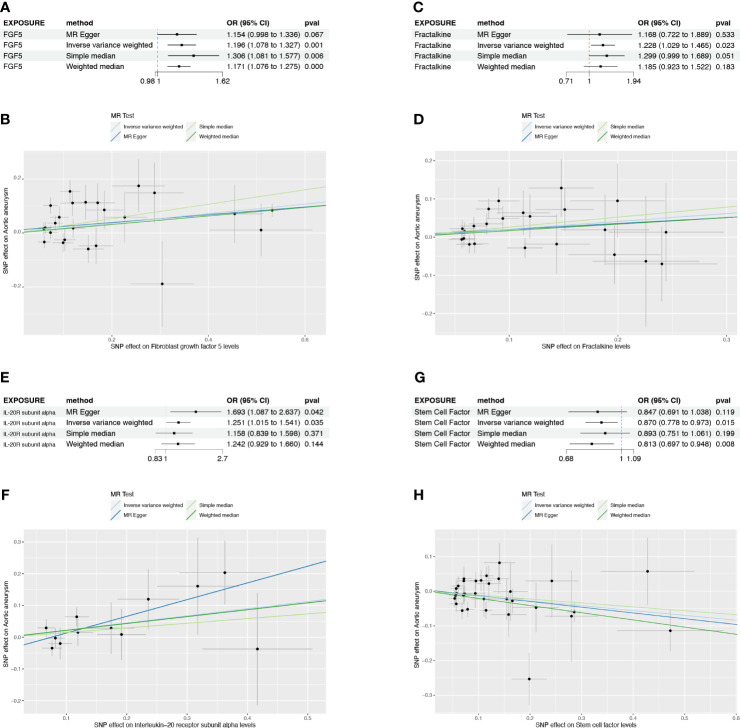
The forest plots and scatter plot demonstrating the association between 4 candidates and AA. **(A)** Forest plot demonstrating the positive correlation between FGF5 and AA. **(B)** Scatter plot illustrating the causal effect of 24 IVs on AA with four MR methods. **(C)** Forest plot demonstrating the positive correlation between Fractalkine and AA. **(D)** Scatter plot illustrating the causal effect of 23 IVs on AA. **(E, F)** The positive correlation between IL-20RA and AA. **(G, H)** The negative correlation between SCF and AA.

None candidates showed significant heterogeneity or horizontal pleiotropy (*P*>0.05) in the sensitivity test except for FGF5 (**Supplementary Table S1**, **Supplementary Figure S1**). CX3CL1 demonstrated a positive correlation solely by the IVW method (OR=1.228, 95%CI=1.029-1.465, *p*=0.023) (**Figure 3C**). IL20RA exhibited positive correlation with an OR=1.251 (95%CI=1.015-1.541, *p*=0.035) (**Figure 3E**). SCF was the only negatively correlated cytokine, which was validated by IVW and weight median method (OR=0.870, 95%CI=0.778-0.973, *p*=0.015; OR=0.813, 95%CI=0.697-0.948, *p*=0.008) (**Figure 3G**). The scatter plots of SNPs associated with each of the four candidates were also demonstrated in **Figure 3**. The funnel plot and forest plot of SNPs were listed in **Supplementary Figure S1**.

### The causal effect of AA on circulating cytokines

**Figure 4:**
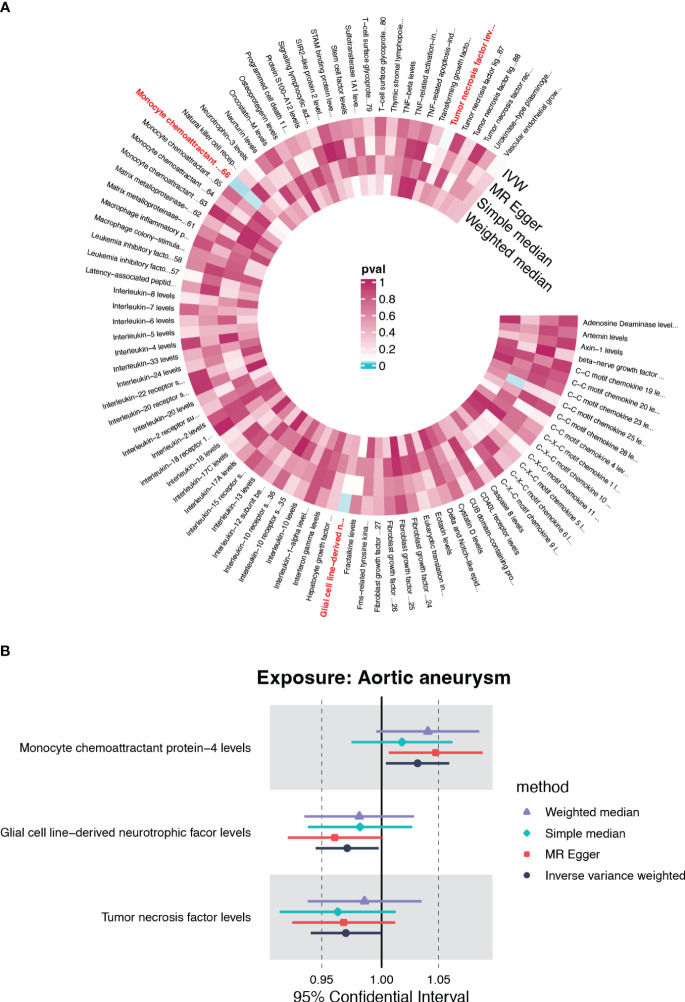
The causal effect of AA on circulating inflammatory cytokines. **(A)** Cyclic heatmap demonstrating p-values for 91 cytokines with each of the four methods when AA was set as exposure. The selectees (*p*<0.05 with IVW) are marked in red **(B)** Forrest plot demonstrating the estimated OR and 95%CI for CCL4, GDNF and TNF as outcome separately.

To further clarify the causal relationships confirmed by the forward analysis, we performed a reverse Mendelian randomization analysis utilizing AA as the exposure. Initial screening using IVW as the primary method suggested that three inflammatory factors, which did not overlap with the four entrants identified in the forward MR analysis, were affected by AA (**Figure 4A**). Of these, monocyte chemoattractant protein-4 (CCL4) was positively regulated by AA (OR=1.031, 95%CI=1.004-1.059, *p*=0.026), while glial cell line-derived neurotrophic factor (GDNF) and tumor necrosis factor (TNF) were negatively regulated by AA (OR=0.971, 95%CI=0.945-0.997, *p*=0.032; OR=0.970, 95%CI=0.941-0.999, *p*=0.046) (**Figure 4B**, **Supplementary Figure S2**, **Supplementary Table S2**). The results of heterogeneity and pleiotropy analysis were demonstrated in **Supplementary Table S2**.

### The expression profile of causal cytokine and corresponding receptor suggested the immunoregulatory role of CX3CR1

**Figure 5:**
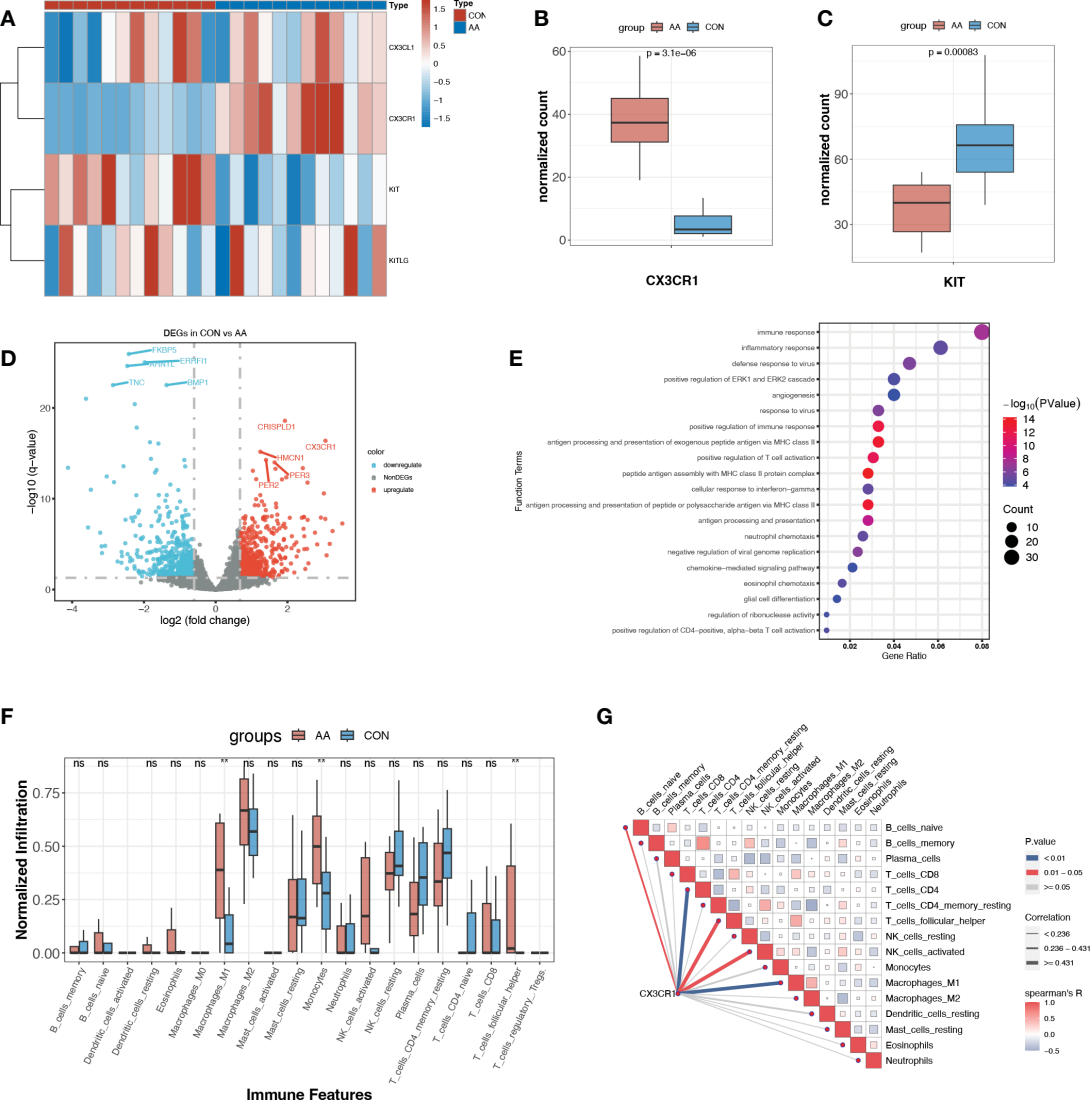
Expression profile of paired ligand-receptor indicated the immunoregulatory role of CX3CR1. **(A)** Heatmap of normalized counts of CX3CL1, CX3CR1, KIT and KITLG. **(B, C)** Normalized counts of CX3CR1 and KIT in AA and control group. **(D)** Volcano plot for DEGs (*p*<0.05, log2FC>0.95) between AA and Control. The top ten most significant DEGs are labeled. **(E)** Gene ontology enrichment analysis of up-regulated DEGs. **(F)** Immune infiltration profile of 20 immune features of AA and control group. **, *p*<0.01. **(G)** Correlation heatmap between CX3CR1 expression and immune features. Blue line, *p*<0.01. ns, P>o.o5 (not significant).

Circulating cytokines generally operate their immune-regulatory function through the interaction with their corresponding receptors. As reported before, CX3CL1 exclusively binds to CX3CR1, which is a 7-transmembrane receptor coupled to heterotrimeric G proteins (GPCR). SCF also cooperates with its exclusive receptor c-kit, which is a tyrosine kinase receptor. The normalized counts of CX3CR1 were significantly up-regulated in AA, while on the contrary, KIT, which encodes c-kit, was significantly down-regulated in AA (**Figures 5A–C**). Their corresponding ligand, CX3CL1, and KITLG showed no significant change between these two groups (**Figure 5A**).

With the criteria of *p*<0.05, log2FC>0.95, we obtained a total of 1120 DEGs, where 513 DEGs were up-regulated and 607 DEGs were down-regulated in AA (**Figure 5C**, **Supplementary Table S3**). Rather surprisingly, we found that CX3CR1 was among the most dramatically up-regulated DEGs in AA (**Figure 5D**). The functional enrichment analysis of the up-regulated DEGs with GO demonstrated that “immune response”, “inflammatory response”, “positive regulation of immune response” and “antigen processing and presentation of exogenous peptide antigen via MHC class II” were among the top 10 enriched terms (**Figure 5E**).

Enlightened by this, we further explored the immune microenvironment utilizing immune infiltration analysis. It turned out that M1 macrophages and monocytes were significantly infiltrated more in AA samples (**Figure 5F**). Another novel finding was that the expression of CX3CR1 was positively correlated with M1 macrophage and CD4^+^ T cell (**Figure 5G**), suggesting its potential role in pro-inflammatory response and adaptive immune response.

### The causal effect of genetically predicted immune cell trait on AA

**Figure 6:**
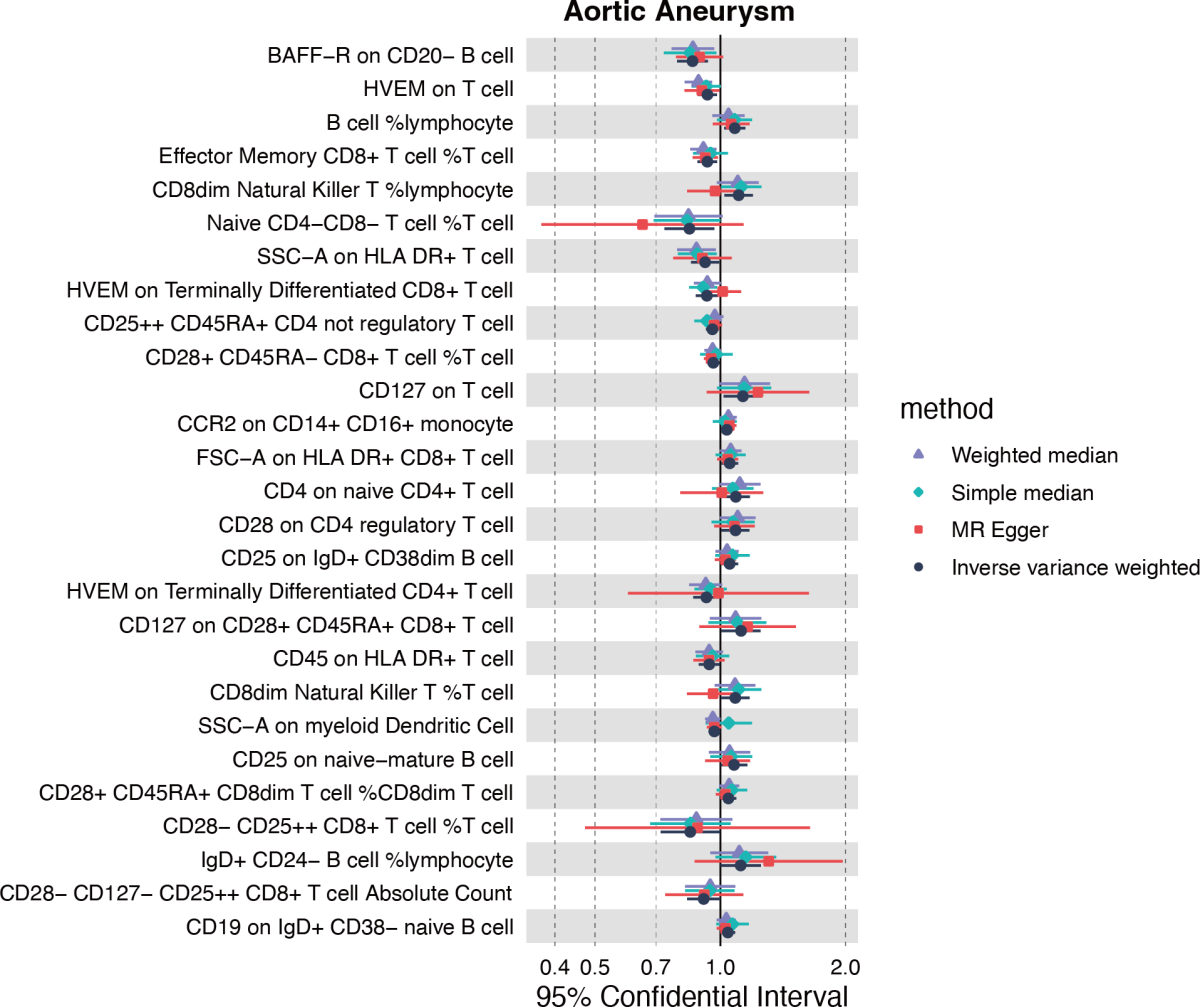
Causality of immune cell traits on AA.

Inspired by the putative role of CX3CR1 in regulating the immune response in AA, especially characterized by the significantly up-regulated infiltration of M1 macrophage, we further explored the causal relationship between immunophenotype and AA at the genetic level. The IVW method was used as the main criteria to explore the causal effect of 731 immunophenotypes on AA. With the threshold of *p*<0.05, we obtained 13 immunophenotypes negatively and 14 immunophenotypes positively correlated with AA (**Figure 6A**, **Supplementary Table S4**).

Among the selectees, we noticed that genetically determined CCR2 on CD14+CD16+ monocytes was positively correlated with the onset of AA (OR=1.036, 95%CI=1.002-1.071, P=0.035) (**Supplementary Table S4**). Furthermore, the MR eggar method demonstrated that CX3CR1 on CD14^+^CD16^-^ monocytes was positively correlated with AA (OR=1.219, 95%CI=1.091-1.362, P=0.003), although no significance was acquired with IVW method (**Supplementary Table S4**). Considering the generally recognized classification of monocytes, CD14 and CD16 were used to distinguish between classical (CD14^+^CD16^-^) and nonclassical (CD14^-^CD16^+^) subtypes, while growing evidence suggested the potential role of CCR2 and CX3CR1 in classifying monocytes in a more detailed manner.

### Expression profile on single cell level identifies the CX3CL1-CX3CR1 signaling between ECs and monocytes

**Figure 7:**
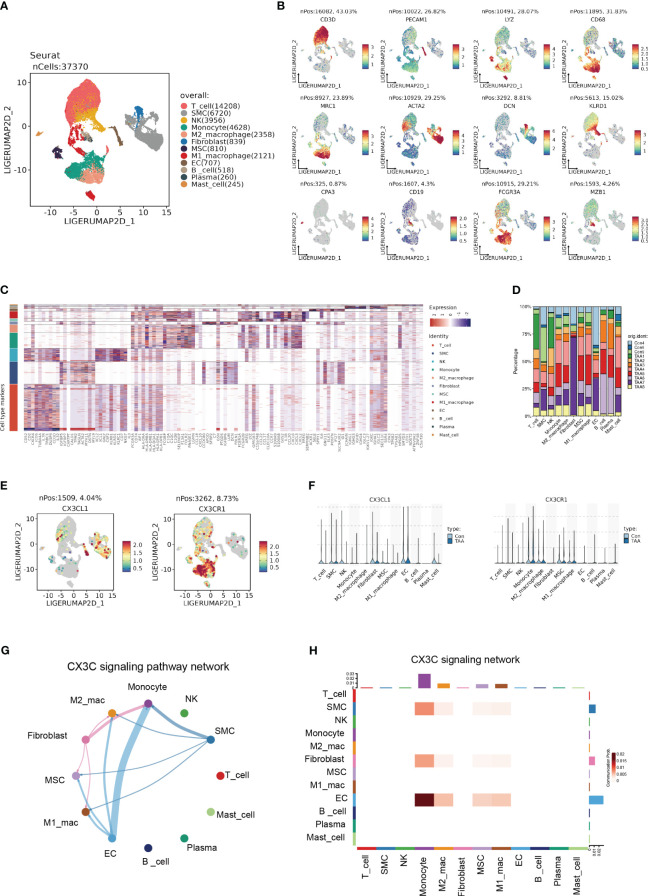
Single cell expression profile identified the CX3C signaling between ECs and monocyte. **(A)** UMAP plot revealing 12 clusters differentiated by different colors. **(B)** Feature plots of corresponding marker genes related with different clusters. **(C)** Mean expression of selected genes in all 12 clusters. **(D)** The percentage of 11 samples in 12 cell types. **(E)** Feature plot demonstrating the location and expression level of CX3CL1 and CX3CR1. **(F)** The expression of CX3CL1 and CX3CR1 among 12 clusters. **(G)** Chord diagram from CellChat analysis revealing CX3C signaling pathway among 12 clusters. **(H)** Heatmap demonstrating the communication probability.

In order to further identify the cell-type localization of the ligand-receptor relationship (CX3CL1-CX3CR1) as indicated above, we re-analyzed the previously published scRNA-seq data from 3 controls and 8 AA samples (3). Similar to the original research, we identified 12 primary cell types, including T cell, smooth muscle cell (SMC), natural killer cell (NK), monocyte, M2 macrophage, fibroblast, mesenchymal stem cell (MSC), M1 macrophage, endothelial cell (EC), B cell, Plasma cell and Mast cell (**Figures 7A–C**). The cell counts of each sample indicated the aggregation of immune cells in AA samples, especially monocytes, M2 macrophages, and M1 macrophages (**Figure 7D**).

Based upon the preliminary clustering, we identified that CX3CL1 was predominantly expressed by ECs and CX3CR1 was mainly expressed by monocytes as demonstrated by the feature plots and violin plots (**Figures 7E, F**). CellChat analysis exhibited an intense signal from ECs to monocytes (**Figures 7G, H**), which indicated that ECs were the major source of CX3CL1 that binds with CX3CR1 on monocytes to leverage downstream effects.

### The CX3CL1-CX3CR1 interaction caused the pro-inflammatory activation of CD14^+^CD16^+^ monocytes

**Figure 8:**
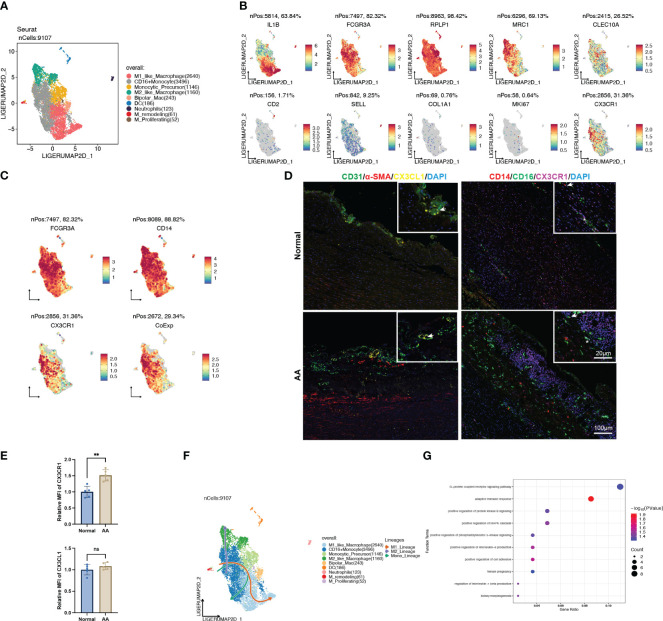
CX3CL1 promoted the pro-inflammatory activation of intermediate monocyte. **(A)** UMAP plot of 9 clusters of myeloid cells differentiated by different colors. **(B)** Feature plots of related marker genes. **(C)** Feature plots of FCGR3A (CD16), CD14 and CX3CR1. The lower-right feature plot reveals the co-localization of all three. **(D)** Multiplex immunofluorescence staining images of normal and ascending aortic aneurysm specimens. Left panel, arrows indicate the co-localization of CX3CL1 and CD31; Right panel, Asterisks indicate the co-localization of CD16 and CX3CR1, while arrows indicate the co-localization of CD16, CD14 and CX3CR1. **(E)** Relative mean fluorescence intensity of CX3CR1 and CX3CL1 in normal and AA samples. N=5; **, *p*<0.01. **(F)** Trajectory analysis of CD16^+^ monocytes revealing 3 potential differentiation lineages. **(G)** Gene ontology enrichment analysis of top 100 DEGs between CX3CL1 treatment and control group.

To further identify the heterogenicity of the myeloid cell lineage, we defined 9 distinct clusters from the original monocyte/macrophage population, consisting of M1-like macrophage, CD16^+^ monocyte, monocytic precursor, M2-like macrophage, bipolar macrophage, dendritic cell (DC), neutrophil, remodeling macrophage and proliferating macrophage (**Figures 8A, B**). Remarkably, the signature genes of CD16^+^ monocyte were enriched in GO terms analogous to DEGs between normal and AA tissue, such as “antigen processing and presentation of exogenous peptide antigen via MHC class II” (**Supplementary Figure S3**).

We noted that the population of cells with high CX3CR1 expression also exhibited high expression of CD14 and CD16 (**Figure 8C**), which was corroborated by colocalized immunofluorescent staining of dilated ascending aorta (**Figure 8D**). Also, we observed co-localization of EC-specific marker CD31 and signaling molecular CX3CL1 (**Figure 8D**). In addition to the normalized count obtained from transcriptomic data (**Figure 5B**), we observed that the expression of CX3CR1 was significantly higher in AA samples compared with normal samples (**Figure 8E**). Trajectory analysis revealed the pluripotency of CD16^+^ monocytes, which could differentiate either to the M1 or M2 polarization status, as well as self-conversion (**Figure 8F**, **Supplementary Figure S3**), whereas the driving force for direction might well be different cytokines.

Thus, we explored the CytoSig database which is composed of transcriptomic profiles of targeted genes modulated by cytokines (14). We obtained the processed microarray data of the primary human CD16^+^ monocytes treated with or without recombinant human CX3CL1 (**Supplementary Table S5**). Through perusal of the sorting protocol, we noticed that these sequenced cells had been positively sorted in advance by CD14. The top 100 up-regulated genes upon CX3CL1 treatment were enriched in the “G-protein coupled receptor signaling pathway”, “adaptive immune response”, “positive regulation of interleukin-8 production” and “positive regulation of cell adhesion” (**Figure 8G**), which all indicated a shift towards the pro-inflammatory state, thus leading to the inflammatory cascade in the pathogenesis of AA.

## Discussion

In this study, we adopted a Mendelian randomization approach to explore the causal relationship between circulating inflammatory cytokines and AA. Coupled with the analysis of bulk RNA sequencing, we identified the CX3CL1-CX3CR1 signaling pathway as a core causative factor for AA and preliminarily established its association with positive regulation of inflammatory response. Further study of the causal association between extensive immunophenotype and AA led us to shift our attention to CD14+CD16+ monocytes, and ultimately, through final validation in single-cell sequencing, we managed to localize the core signaling pathway to the cellular level and finally fine-tuned a pro-inflammatory signaling pathway form EC to monocyte mediated by CX3CL1-CX3CR1 in AA.

Mendelian randomization is credited as nature’s randomized trial, where genetic alleles are taken as the variant, conquering some biases in standard observational studies (11). This approach has been used to confirm the causality of elevated LDL, decreased HDL, and AA (15), in addition to elucidating the drug targets for AA (16). Our study was the MR study on inflammation and AA with the most variables included so far. The GWAS summary data we obtained was derived from a cross-population meta-analysis containing European and East Asian ancestors (8), which further bolstered the generalizability of our conclusion. However, the overbroad AA phenotypes included were not differentiated between thoracic or abdominal aortic aneurysm, and the known inherited fractions (Marfan syndrome, Loeys-Dietz syndrome, bicuspid aortic valve, etc.) (17) were not analyzed separately, which was detrimental to precise medical decisions going forward.

CX3CL1, alternatively referred to as fractalkine, is the most specialized member of chemokines owing to its membrane-bound and secreted form, acting as a recruiter and adherent respectively (18). Serum CX3CL1 level was found positively associated with atherosclerotic cardiovascular disease and higher levels of inflammatory cytokines (19). Back in 2008, it was suggested that CX3CL1 might accelerate AA progression through recruiting inflammatory cells. Our study resolved the unidirectional causal relationship between CX3CL1 and AA for the first time with genetic tools, further suggesting the great potential of CX3CL1 as the predictor for AA. ECs exposed to disturbed flow upregulated CX3CL1 (20), and the neutralizing antibody against it significantly alleviated the aortic lesion area in ldlr knockout mice (21), suggesting its potential as an intervention target.

CX3CR1 is the exclusive receptor for CX3CL1, although it was suggested that CCL26 was also a functional ligand for CX3CR1, the affinities of these two were vastly divergent (22). The binding capacity between CX3CL1 and CX3CR1 is strong enough to allow rapid infiltration into the vessel wall independent of classical adhesion molecules such as intracellular adhesion molecular (ICAM)-1 to perform immune surveillance (23), which is one of the reasons for endowing CX3CR1+ monocytes with the name “patrollers”. In mice, two monocyte populations have been characterized: Ly6C^hi^CCR2^+^CX3CR1^int^ classical monocytes and Ly6C^lo^CCR2^-^CX3CR1^hi^ nonclassical monocytes (24). But even before relying on CX3CR1 for functional categorization, human circulating monocytes were first divided into the classical (CD14^+^CD16^-^), nonclassical (CD14^-^CD16^+^) and intermediate (CD14^+^CD16^+^) (25). The remarkable co-expression of CD16 and CX3CR1 found in our study partly illustrates the cross-species consistency of this classification. Although we noted a significant infiltration of intermediate monocytes in AA based on scRNA-sequencing data, it is fundamentally different from traditional protein-expression-based clustering, due to which this conclusion requires further validation. Also, our pseudotime analysis actually revealed multiple plasticities of these monocytes, however, the validation of downstream effects was entirely reliant on one independent study, which might bring bias to the conclusion. Despite numerous studies confirming the long-acting pro-inflammatory role of CX3CL1-CX3CR1 in cardiovascular disease (26, 27), and the antagonist of CX3CR1 having been subjected to preliminary clinical translation (28, 29), considering the recently demonstrated protective role of this axis in neurodegenerative disease (30), a large number of *in vitro* experiments are still in need for subsequent validation.

In conclusion, the present study validated the pathogenic role of CX3CL1-CX3CR1 in AA, and targeted inhibition might alleviate the progression of AA.

## Data availability statement

‘The original contributions presented in the study are included in the article/**Supplementary Materials**, further inquiries can be directed to the corresponding author/s.

## Ethics statement

The studies involving humans were approved by Wuhan Union Hospital, Huazhong University of Science and Technology (UHCT22975). The studies were conducted in accordance with the local legislation and institutional requirements. The participants provided their written informed consent to participate in this study.

## Author contributions

XQ: Writing – original draft, Writing – review & editing. YZ: Writing – original draft, Writing – review & editing. LX: Writing – review & editing. ZL: Writing – review & editing. MC: Writing – review & editing. FT: Writing – review & editing. PF: Writing – review & editing. ZC: Writing – review & editing. ND: Writing – review & editing. CZ: Writing – review & editing. JL: Writing – review & editing.

## Supplementary material

Supplementary Figure 1 Funnel plots and forest plots of leave-out-one analysis when AA was set as outcomes. **(A, B)** IVs of FGF5. **(C, D)** IVs of Fractalkine. **(E, F)** IVs of IL20RA. **(G, H)** IVs of SCF.

Supplementary Figure 2 Forest plots, scatter plots and funnel plots when AA was set as exposure. **(A–D)** GDNF as outcome. **(E–H)** CCL4 as outcome. **(I–L)** TNF as outcome.

Supplementary Figure 3 Analysis of myeloid cluster. **(A)** Expression of selected marker gene of each cluster. **(B)** GO and KEGG enrichment analysis of marker genes of each cluster. **(C)** Feature plots of trajectory analysis demonstrating 3 potential lineages.
