# Supplementary figures and images for: Deciphering the role of CX3CL1-CX3CR1 in aortic aneurysm pathogenesis: insights from Mendelian randomization and transcriptomic analyses
Source: Front Immunol. 2024 Apr 23;15:1383607. doi: 10.3389/fimmu.2024.1383607 (PMC11074460; doi:10.3389/fimmu.2024.1383607)

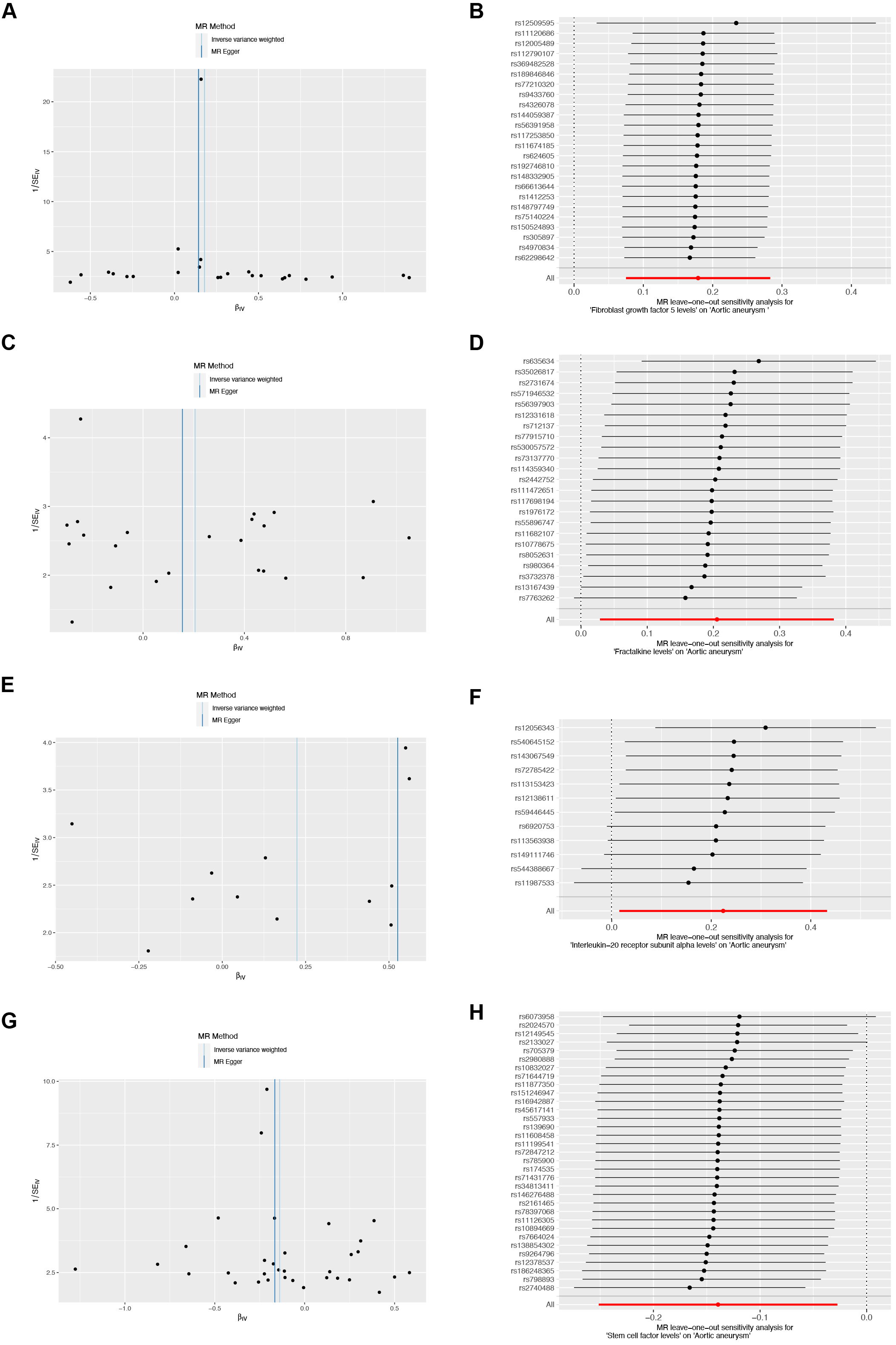

Supplement: Supplementary Figure 1 — Funnel plots and forest plots of leave-out-one analysis when AA was set as outcomes. (A, B) IVs of FGF5. (C, D) IVs of Fractalkine. (E, F) IVs of IL20RA. (G, H) IVs of SCF. [file Image_1.jpeg]

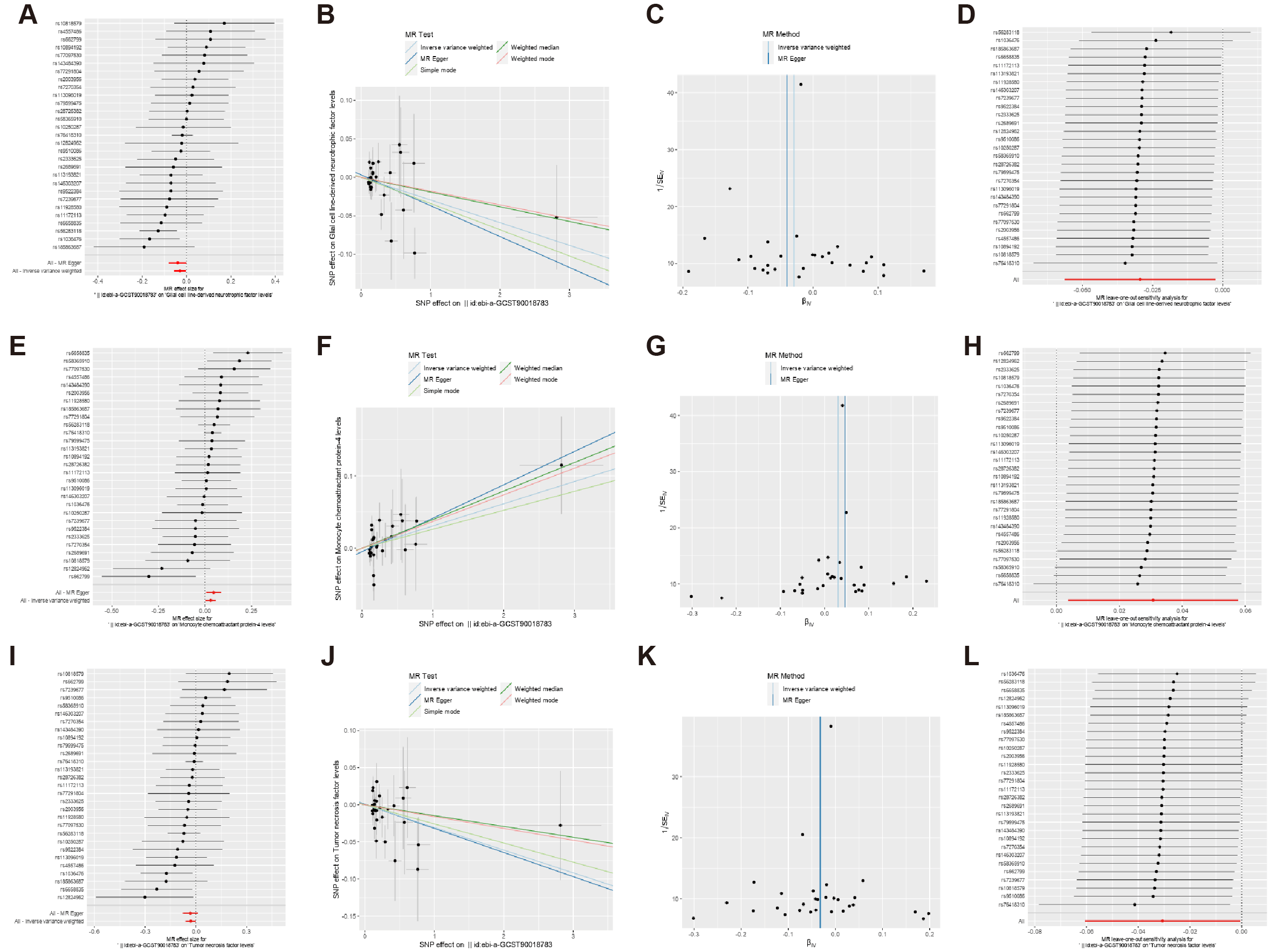

Supplement: Supplementary Figure 2 — Forest plots, scatter plots and funnel plots when AA was set as exposure. (A–D) GDNF as outcome. (E–H) CCL4 as outcome. (I–L) TNF as outcome. [file Image_2.jpeg]

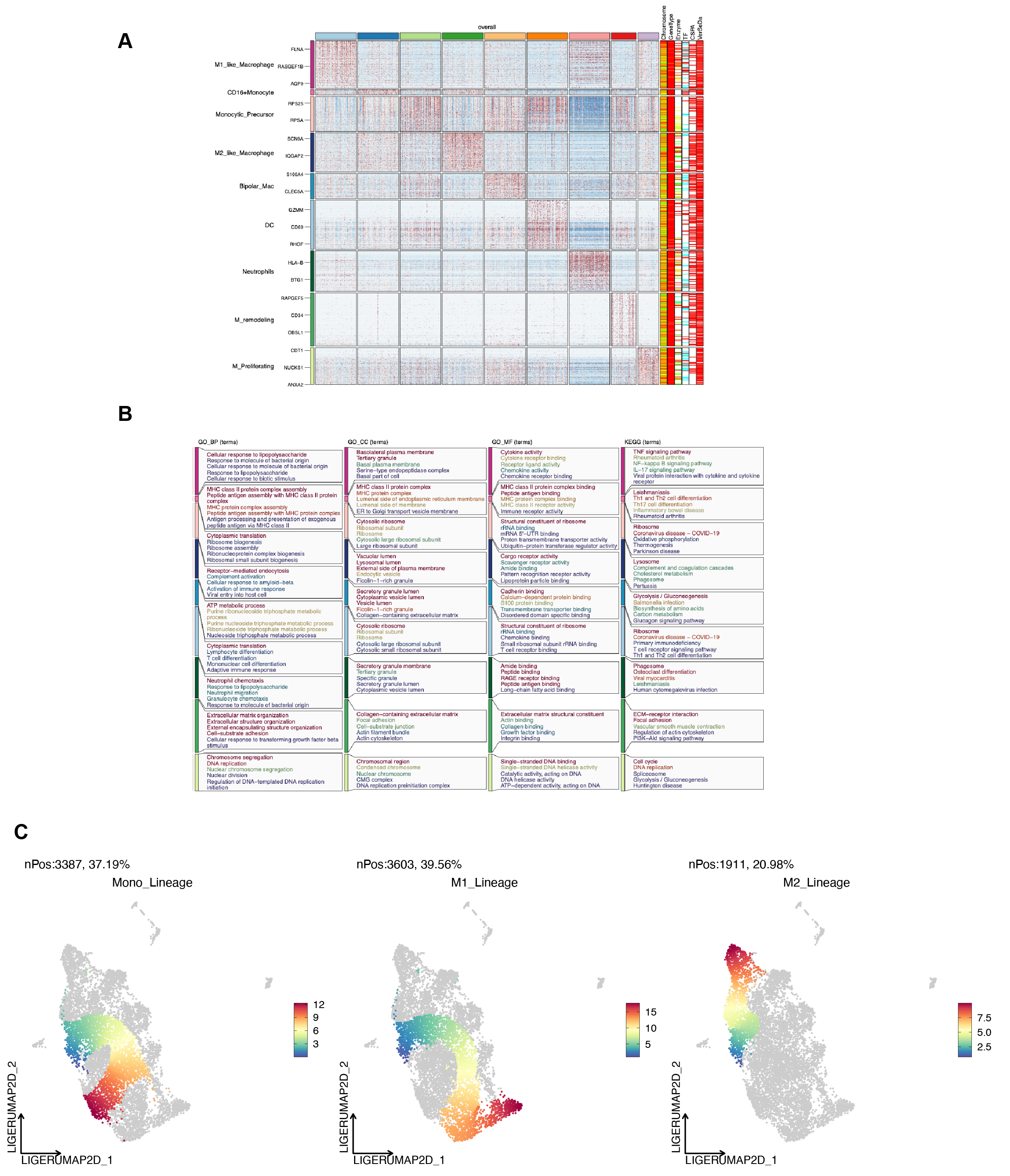

Supplement: Supplementary Figure 3 — Analysis of myeloid cluster. (A) Expression of selected marker gene of each cluster. (B) GO and KEGG enrichment analysis of marker genes of each cluster. (C) Feature plots of trajectory analysis demonstrating 3 potential lineages. [file Image_3.jpeg]
